# Fatty Acid Profile of Table Grapes: Impact of Cultivar and Fruit Protection on Saturated, Monounsaturated, and Polyunsaturated Fatty Acid Profile

**DOI:** 10.3390/foods15091585

**Published:** 2026-05-04

**Authors:** Nataly Tatiana Coronel Montesdeoca, Lucía Andreu-Coll, Guillermo Alexander Jácome Sarchi, Francisca Hernández, Esther Sendra

**Affiliations:** 1 Grupo de Investigación Agricultura Sostenible (GIAS), Carrera de Agropecuaria, Universidad Politécnica Estatal del Carchi, Tulcán 040102, Ecuador; guillermo.jacome@upec.edu.ec; 2 Grupo de Fruticultura, Departamento de Producción Vegetal y Agrotecnología, Instituto Murciano de Investigación y Desarrollo Agrario y Medioambiental (IMIDA), Calle Mayor, s/n, 30150 La Alberca, Murcia, Spain; lucia.andreu@carm.es; 3 Grupo de Investigación en Fruticultura y Técnicas de Producción, Instituto de Investigación e Innovación Agroalimentaria y Agroambiental (CIAGRO-UMH), Universidad Miguel Hernández, Carretera de Beniel, km 3,2, 03312 Orihuela, Alicante, Spain; 4 Grupo de Investigación en Calidad y Seguridad Alimentaria, Instituto de Investigación e Innovación Agroalimentaria y Agroambiental (CIAGRO-UMH), Universidad Miguel Hernández, Carretera de Beniel, km 3,2, 03312 Orihuela, Alicante, Spain; esther.sendra@umh.es

**Keywords:** *Vitis vinifera* L., lipidome remodeling, thermal stress adaptation, cardioprotective lipids, atherogenicity index, agricultural microclimate

## Abstract

Pre-harvest bagging protects table grapes from environmental stress, yet its interannual impact on the plant fatty acid profile remains underexplored. This study aimed to evaluate the fatty acid profile and Atherogenicity Index (AI) and Thrombogenicity Index (TI) as health indices of three traditional cultivars (“Doña María”, “Dominga”, “Aledo”). Grapes were cultivated under bagged and unbagged conditions over two consecutive seasons characterized by contrasting thermal stress, and their lipid extracts were evaluated using gas chromatography and multivariate statistical tools. The analytical results revealed a profile predominantly composed of polyunsaturated fatty acids, primarily linoleic acid. Both genotype and protective bagging significantly modulated lipid accumulation. Uniquely, the extreme heatwave of the second season triggered a profound lipid remodeling, increasing the total polyunsaturated fraction by over 40% in “Doña María” as a putative adaptive response to maintain membrane fluidity. Nutritionally, all cultivars demonstrated exceptional cardioprotective potential, recording atherogenicity indices below 0.11. These findings provide novel insights into the resilience of table grapes, validating them as a robust source of functional lipids. Furthermore, tracking this lipid remodeling offers the agricultural industry a sensitive biochemical tool to optimize protective strategies and ensure crop quality under increasing climate volatility.

## 1. Introduction

*Vitis vinifera* L. is currently defined not only as an agricultural product of great economic importance, but also as a functional food and a valuable source of nutraceuticals due to its bioactive compounds [1,2]. The nutritional composition of table grapes is predominantly associated with high concentrations of carbohydrates (such as glucose and fructose) and antioxidant polyphenols [3,4,5]. However, despite constituting a minority fraction—representing approximately 0.15% to 1% of the berry’s fresh weight—the lipid profile plays an indispensable physiological and nutritional role [6].

These lipids, composed mainly of polyunsaturated and monounsaturated fatty acids (MUFA) such as linoleic and oleic acid [1], are critical for cell integrity, as they form the fundamental basis of biological membranes and regulate essential physiological functions [2,7]. For consumer health, the intake of these fatty acids is vital, as they act as precursors in the formation of neuronal and retinal membranes, participate in cell signaling, and offer protection against oxidative stress and apoptosis [1].

The nutritional and functional quality of these lipids relies heavily on the polyunsaturated to saturated fatty acids (PUFA/SFA) ratio and the omega-6/omega-3 balance. While excess SFAs are linked to metabolic dysfunctions, PUFAs are essential for cardiovascular health [8,9]. To accurately assess this cardioprotective potential beyond simple quantification, advanced functional metrics such as the Atherogenicity Index (AI) and Thrombogenicity Index (TI) are employed. These indices evaluate the capacity of lipids to inhibit plaque aggregation and clot formation, providing a robust, multidimensional measure of the fruit’s functional quality [2,10].

The commercial quality and bioactive profile of table grapes depend not only on genetic factors, but also on pre-harvest management practices. Among these, bagging has established itself as a fundamental strategy for the physical protection of the bunch against pests, diseases, and environmental stress, while significantly reducing the need to apply agrochemicals [11,12,13]. In addition to its protective function, bagging drastically modifies the microclimate around the berry by altering temperature, humidity, and sunlight exposure, which directly impacts its secondary metabolism and phenological development [14,15].

In this context, in varieties under the Protected Designation of Origin (PDO) “Uva de Mesa Embolsada del Vinalopó” (bagged Table Grapes from Vinalopó), such as “Aledo,” “Dominga,” and “Doña María,” it has been observed that the profile of volatile compounds varies significantly depending on the cultivar and the application of this protection technique. Previous studies indicate that while “Aledo” bunches stand out for their abundance of more than 80% aldehydes, the mid-season variety “Doña María” maintains a higher proportion of terpenes under bag protection [16].

However, the relevance of these findings lies in the fact that most of these volatile compounds, particularly six-carbon (C6) aldehydes such as hexanal, are derived directly from the enzymatic degradation of PUFA (linoleic and linolenic) via the lipoxygenase pathway.

Therefore, bagging not only acts as a physical barrier against pests and environmental stresses, but also modifies the thermal and light microclimate of the berry, potentially altering the lipid reserve that serves as a substrate for aroma formation and membrane integrity during ripening [16]. Although it has been established that bagging in the Vinalopó Valley preserves a fresher aromatic profile by delaying the senescence processes [16], there is a critical gap in the literature regarding how this technique affects the functional nutritional quality of fatty acids and cardiovascular health indices (AI and TI). Furthermore, considering that table grapes are not traditionally recognized as a primary source of dietary lipids, the comprehensive characterization of their fatty acid profile transcends purely nutritional and functional interests. This specific fatty acid fingerprint can serve as a highly sensitive molecular marker that reflects the impact of specific pre-harvest agronomic practices. Consequently, profiling these lipids—in conjunction with the previously discussed shifts in volatile compounds—provides a powerful, multidimensional tool to guarantee the authenticity, traceability, and differential quality of these Protected Designation of Origin (PDO) varieties and their unique cultivation methods. This gap is particularly noticeable in studies covering multiple campaigns, as the interannual stability of these lipid profiles in the face of climatic variability has not been reported exhaustively.

This literature gap is particularly critical when considering interannual climatic volatility, as the stability of these lipid profiles across contrasting campaigns remains underexplored. While bagging in the Vinalopó Valley effectively delays senescence and preserves volatile compounds [16], the Mediterranean basin is increasingly subjected to severe thermal extremes. According to the Spanish State Meteorological Agency (AEMET), the July–August 2023 period was the hottest on record for the Valencian Community since 1950. The Vinalopó region endured unprecedented heatwaves, with maximum temperatures frequently exceeding 42 °C peaking at 44.3 °C in the Vinalopó area during the August extreme thermal event, coupled with a historic record of torrid nights [17]. There is a critical gap in the literature regarding how these extreme thermal anomalies interact with the bagging microclimate to alter the plant’s lipid reserve, which serves both as a structural defense mechanism against heat stress and as a substrate for post-harvest aroma formation.

Consequently, this research addresses this gap through a detailed characterization of the fatty acid profile in the “Aledo,” “Dominga,” and “Doña María” varieties. The study evaluates the impact of pre-harvest bagging on the fatty acid profile and functional nutritional quality during two consecutive and climatically contrasting production cycles (2022–2023).

## 2. Materials and Methods

### 2.1. Plant Material and Sampling Protocol

For this research, table grape varieties (*Vitis vinifera* L.) with different ripening cycles and organoleptic profiles grown in the Vinalopó Valley, Alicante, Spain, were selected. The study was conducted on adult table grape vines (approximately 10 years old) grafted onto 140 Ruggeri (140 Ru) rootstocks, recognized for their drought resistance and vigor. The vines were trained on a traditional trellis system (*espaldera*) with a planting framework of 2.0 × 2.5 m.

The varieties studied were “Doña María,” which is a mid-season variety harvested between September and October. It is characterized by large, loose clusters with pale yellow berries, thin skin, and firm, juicy flesh that stands out for its characteristic honeyed aroma. The “Aledo” variety is a late-ripening variety harvested between November and December; it has large, loose clusters with ellipsoidal, waxy yellow berries protected by thick, crunchy skin. The “Dominga” variety was evaluated during the 2022 cycle as a benchmark for early to mid-ripening in October–November. It has large clusters with uniform, elongated, straw-yellow berries, firm flesh, and a neutral flavor [16].

The harvest was carried out manually during two consecutive seasons (2022 and 2023), following commercial maturity criteria (uniformity of color and berry size). The harvest schedule was adjusted to the phenological cycle of each variety. The “Doña María” variety was harvested in the second week of October (mid-season cycle) and the ‘Dominga’ and “Aledo” varieties were harvested in the last week of November (late cycle). It is important to note that the “Dominga” variety was evaluated solely during the 2022 cycle as a benchmark for early to mid-ripening. It was excluded from the 2023 season analysis because the required pre-harvest bagging practices were not performed by the grower in accordance with the strict Protected Designation of Origin (PDO) Vinalopó regulatory standards. Consequently, these samples did not meet the legal quality requirements of the designation, and their inclusion would have compromised the inter-annual comparability and scientific integrity of the experimental results.

### 2.2. Location and Agroclimatic Conditions

The study was conducted on commercial farms located in Novelda (Alicante, Spain; coordinates: UTM ETRS89 30N, X: 695138, Y: 4252647), within the production area of the PDO “Uva de Mesa Embolsada del Vinalopó”. The region has a strictly Mediterranean climate, characterized by mild winters, low annual rainfall, and hot, dry summers.

To properly contextualize the experimental period within the region’s long-term climatology, a 30-year historical database (1991–2020) from the Spanish State Meteorological Agency (AEMET) [17] was analyzed. According to these historical records, the crucial ripening period from August to October is characterized by standard Mediterranean temperature and precipitation patterns, with average monthly temperatures ranging from 26.4 °C in August to 20.0 °C in October, and historically low summer rainfall (a historical average of 10.2 mm for August). The 2022 season closely followed these standard historical conditions. In stark contrast, the 2023 season represented a statistically extreme anomaly. The average monthly temperature for August 2023 increased to 27.3 °C, significantly exceeding the 30-year historical average. Furthermore, this period was subject to unprecedented heat waves, with maximum temperatures reaching 44.3 °C in the Vinalopó area. Coupled with a severe rainfall deficit of 36% during August compared to the already low historical average of 10.2 mm, this contrasting interannual climatology provided a very robust natural experiment for evaluating the metabolic plasticity of grapevines under severe climate change scenarios, fully justifying the experimental design.

### 2.3. Experimental Design and Bagging Treatment

A factorial experimental design was applied to evaluate the impact of the fruit protection technique. In August 2022 and 2023, specifically at the BBCH 81 phenological stage (veraison: beginning of ripening, when berries begin to brighten), grape clusters were individually protected using bags. In strict compliance with the Vinalopó PDO regulatory standards, these bags are made of long-fiber cellulose treated with melamine for moisture resistance, featuring a satin finish, a characteristic cane color, and a standard basis weight of 40 g/m^2^.

Control plots (unbagged clusters) were selected within the exact same experimental orchards and on adjacent vines under identical growing conditions during both consecutive seasons. To ensure that the microclimate generated by the bag was the only differentiating variable, all treatments (bagged and uncovered controls) within the same cultivar were subjected to identical agronomic management, receiving the exact same drip irrigation volumes and fertilization programs throughout the study.

### 2.4. Sample Preparation and Selection Criteria

Once in the laboratory, a thorough inspection of the 30 clusters for each combination of variety and treatment was carried out. To ensure robust statistical and biological representativeness that accounts for inherent inter-cluster and intra-cluster variability, a pooled sampling strategy was employed. Specifically, approximately 10 berries were manually selected from each of the 30 clusters (totaling a representative pool of ~300 berries per biological replicate). These berries were systematically excised from the upper, equatorial, and lower sections of the rachis to mitigate developmental gradients and microclimatic differences (such as differential sunlight exposure and ripening rates) within the cluster. Strict exclusion criteria were applied to ensure data integrity; only intact berries were included, discarding those that showed dehydration, mechanical damage, or signs of fungal infection.

To specifically isolate the lipid profile of the exocarp and mesocarp, the seeds were manually removed from all selected berries. Subsequently, in order to stop any endogenous enzymatic activity and prevent the oxidation of PUFAs, the seedless tissue was divided into three biological replicates per treatment (approximately 40 g fresh weight per replicate). Each replicate was immediately frozen and homogenized using liquid nitrogen (−196 °C). The cryotriturated plant material was fractionated into aliquots and stored at −80 °C until subsequent lyophilization and lipid extraction.

To evaluate the potential protective effect of the lipid fraction against coronary heart disease, the Atherogenicity Index (AI) and Thrombogenicity Index (TI) were calculated. These mathematical models, although classic, have recently been validated as robust tools for determining nutritional quality in a wide range of vegetable matrices and seed oils, allowing for objective classification of the health potential of food [18,19]. Likewise, the hypocholesterolemic/hypercholesterolemic (HH) ratio was used to complement the evaluation, following standardized protocols for plant-based lipids in advanced nutrition studies [20,21].

### 2.5. Lipid Analysis

Fatty acid methyl esters (FAMEs) were prepared for lipid profiling according to the ISO-12966-2 standard, adapting the protocols previously described by Andreu-Coll et al. [22] and Trigueros et al. [23]. A sequential alkaline and acid transmethylation/methylation procedure was followed. Briefly, an initial alkaline methylation was performed using sodium methoxide under reflux for 10 min at 90 °C, followed by an acid methylation with boron trifluoride for 25 min at room temperature in the dark. Tridecanoic acid (C13:0; 0.04 mg mL^−1^). All reagents used were from Supelco, Sigma-Aldrich (Bellefonte, PA, USA) was added to the samples as an internal standard for precise quantification.

Separation and quantification were performed using a Shimadzu GC-2030 gas chromatograph equipped with a flame ionization detector (FID) and an AOC-20i automatic injector, following the analytical framework described by García-Garví et al. [24]. The fatty acids were separated using a Supelco SP^®^-2380 capillary column (length: 60 m, internal diameter: 0.25 mm, and film thickness: 0.20 µm, Supelco, Sigma-Aldrich (Bellefonte, PA, USA). The equipment conditions were as follows: injector and detector temperatures were set at 250 °C and 260 °C, respectively. Samples were injected at a 1:20 split ratio, using helium as the carrier gas at a linear flow velocity of 28.4 cm s^−1^. The oven temperature started at 70 °C and increased up to 250 °C at a rate of 3 °C min^−1^. The analyses were performed in triplicate. Methyl fatty acids were identified by comparison of retention times with the FAME Supelco MIX-37 standards (Supelco, Sigma-Aldrich, Bellefonte, PA, USA), and the results were expressed in mg kg^−1^ of dry weight (DW).

Additionally, the health potential of the lipid fraction was evaluated by calculating the atherogenic index (AI), the thrombogenic index (TI), and the hypocholesterolemic/hypercholesterolemic ratio (HH), as well as the ratio between linoleic acid and α-linolenic acid (LA/ALA ratio). These nutritional indices were calculated to determine whether the lipidome remodeling induced by pre-harvest bagging and interannual thermal stress significantly impacts the cardioprotective potential and functional value of the table grapes for human consumption, thereby linking agronomic management to fruit quality. The AI, TI, and HH indices were calculated using the equations proposed by Ulbricht and Southgate [25] and Chen and Liu [26], while the LA/ALA ratio was determined as a direct nutritional indicator of the omega-6/omega-3 structural balance.

### 2.6. Statistical Analysis

The experimental data were subjected to analysis of variance (two-way ANOVA) to evaluate the effect of the main factors Variety (V) and Treatment (T) as well as their interaction (V × T) on the lipid profile and health indices. In cases where significant differences were detected, Tukey’s multiple range test was applied for the separation of means.

All levels of statistical significance were set at *p* < 0.05, *p* < 0.01, and *p* < 0.001. The results are presented as the mean of three biological replicates. Data processing and statistical analyses were performed using R-Studio software (version 2023.06.0+421).

## 3. Results

**Figure 1:**
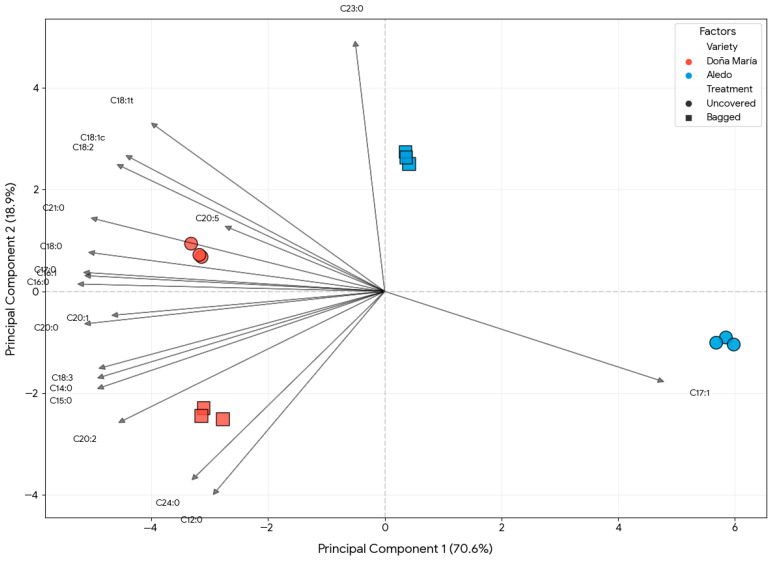
PCA Biplot of the fatty acid profile of table grapes (“Doña María” and “Aledo”) during the 2023 campaign. The plot illustrates the distribution of samples based on the interaction between cultivar and bagging treatment. Vectors represent the loadings of individual fatty acids, indicating their contribution to the separation along Principal Components 1 and 2 (PC1 and PC2).

To obtain a comprehensive view of the alterations in lipid metabolism induced by genotype and cultural management, a Principal Component Analysis (PCA) was performed on the data set from the 2023 campaign (Figure 1). The first two principal components explained 92.1% of the total variance (PC1: 76.4%; PC2: 15.7%), demonstrating high statistical robustness in the differentiation of samples. The exact numerical contributions of each individual fatty acid to these principal components are detailed in Supplementary Table S1 (PCA Loadings Matrix).

The analysis revealed that genetic background was the primary driver of lipidomic variance. PC1 sharply discriminated the samples by variety: the “Doña María” cultivar was characterized by a PUFA-enriched profile (specifically linoleic and α-linolenic acids), whereas the “Aledo” variety exhibited a distinct, more simplified lipid signature.

Furthermore, PC2 captured the secondary variance driven by agronomic management. A clear separation was observed between the control and bagged fruits within each cultivar, indicating that bagging modifies the lipid reserve through genotype-dependent metabolic shifts. For instance, in “Doña María,” direct environmental exposure (unbagged control) was closely associated with compounds tentatively identified as EPA (C20:5) and palmitoleic acid (C16:1). Conversely, in “Aledo,” the bagging treatment induced a metabolic shift towards a higher concentration of long-chain SFAs (C20–C24).

Overall, the clear clustering confirms that the table grape lipid profile is significantly modulated not only by its genetic origin but also by its history of environmental exposure and agronomic protection during ripening.

Analysis of variance (Table 1) revealed that the composition of SFA was significantly influenced by grape variety, cultivation treatment, and the interaction between both factors. Palmitic acid (C16:0) was identified as the major component within this fraction, showing significant differences (*p* < 0.05) between cultivars. The “Dominga” variety recorded the highest concentration of this compound, ranking above “Doña María” and more markedly above “Aledo”, which had the lowest levels. As for stearic acid (C18:0), highly significant variations (*p* < 0.001) were detected, with “Dominga” again standing out presenting value almost double those observed in the other two varieties. Regarding minor and trace saturated compounds (such as behenic acid, C22:0), their presence was minimal and varied depending on the cultivar. Although these ultra-minority compounds are present in trace amounts within the dry matter and lack direct nutritional relevance, their detection is consistent with recent fatty acid profiling of grape pomace and seeds across different varieties.

Similarly, within the monounsaturated fraction, trace compounds like erucic acid (C22:1) were detected exclusively in the “Doña María” variety at very low concentrations (<9 mg kg^−1^). While statistically significant (*p* < 0.001) in relation to the interaction between genotype and bagging, these trace levels reflect genotypic secondary metabolism rather than a functional nutritional contribution.

Regarding the impact of cultural management techniques, bagging treatment induced a generalized reduction in SFA accumulation compared to exposed clusters. Specifically, palmitic acid decreased by 14.7% in bag-protected fruits, from an average of 5023 mg kg^−1^ to 4285 mg kg^−1^. This reduction pattern was consistent for stearic acid and arachidic acid (C20:0), both with a significance of *p* < 0.001 in favor of the uncovered treatment. Analysis of the Variety × Treatment interaction highlighted that the combination of the “Dominga” variety under direct exposure accumulated the highest saturated lipid content, reaching 6041 mg kg^−1^ of palmitic acid and 2955 mg kg^−1^ of stearic acid.

The fraction of MUFAs was qualitatively dominated by oleic acid (C18:1 c9/n9), which represented the most abundant individual lipid component after PUFAs. Significant differences (*p* < 0.05) were observed depending on the cultivar, with “Dominga” leading production, followed by ‘Aledo’ and finally “Doña María”. As with SFAs, the uncovered treatment favored a significantly higher concentration of oleic acid compared to the bagged treatment.

Other minor compounds also showed specific behaviors of great taxonomic and biochemical interest. Palmitoleic acid (C16:1), although it did not vary significantly between varieties, showed a clear dependence on treatment, accumulating at higher levels in unbagged fruits. On the other hand, erucic acid (C22:1) was exclusively detected in the “Doña María” variety. The interaction between factors was highly significant for the latter compound (*p* < 0.001), indicating that its synthesis is genotype-dependent and is altered by the light and temperature conditions modified by bagging.

While the overall accumulation of total PUFAs exhibited distinct upward trends in specific cultivars like ‘Doña María’ under thermal stress, the individual fatty acids within this group demonstrated highly dynamic and contrasting behaviors depending on the genotype and bagging treatment. For instance, analysis of the polyunsaturated fraction (Table 2) revealed that linoleic acid (C18:2 n6c) was the most abundant individual lipid component in all crops analyzed, representing the largest proportion of the total lipid profile. Significant differences (*p* < 0.05) were observed between varieties, with “Dominga” recording the highest concentration (57,593 mg kg^−1^), followed by “Doña María” (47,740 mg kg^−1^) and “Aledo” (42,797 mg kg^−1^). Regarding α-linolenic acid (C18:3 n3), the results showed highly significant variability (*p* < 0.001), with “Doña María” standing out with the highest levels (370 mg kg^−1^), representing a 45% difference from the minimum levels observed in “Aledo.” A finding of great taxonomic relevance was the tentative detection of EPA, C20:5 n3 in the “Doña María” variety (165 mg kg^−1^), whereas at such retention time no signal was detected in the other two cultivars.

With regard to treatment, bagging significantly reduced the content of most PUFAs compared to exposed clusters. Specifically, linoleic acid decreased from 52,849 mg kg^−1^ in the control to 45,904 mg kg^−1^ in the protected clusters, while α-linolenic acid showed a 14% reduction under the bag treatment. The Variety × Treatment interaction was highly significant for the tentative EPA (*p* < 0.001), with the combination of “Doña María” without bagging reaching the highest concentration (272 mg kg^−1^), while the bagging treatment drastically reduced this value to 58.0 mg kg^−1^, suggesting a strong sensitivity of this compound to light and temperature conditions inside the bag.

In terms of total accumulation (Table 3), the “Dominga” variety recorded the highest total fatty acid content, significantly exceeding “Doña María” and “Aledo.” This trend was maintained in the individual sums of ∑ SFA, ∑ MUFA, and ∑ PUFA, where “Dominga” showed statistical superiority (*p* < 0.05) in all fractions. Regarding the impact of cultural management, the uncovered treatment favored greater total lipid accumulation compared to the bagged treatment, suggesting that direct exposure to solar radiation promotes lipid biosynthesis in the exocarp of the berry.

Despite differences in absolute concentrations, nutritional quality indices revealed an exceptionally healthy profile for all varieties. The AI and TI remained at minimal levels, with no significant differences between cultivars or treatments. However, the Hypocholesterolemic/Hypercholesterolemic (HH) ratio did show notable variations, with the “Aledo” variety having the most favorable ratio, followed by “Doña María”. Finally, the linoleic acid/α-linolenic acid (LA/ALA) ratio showed extreme disparity, with a ratio of 136 in “Doña María” and 212 in “Aledo,” indicating a significantly higher proportion of omega-6 in the latter cultivar.

In the 2023 campaign, the saturated fatty acid profile (Table 4) was marked by a highly significant interaction (*p* < 0.001) between variety and treatment, especially in the major compounds. Palmitic acid (C16:0) consolidated its predominance, registering the highest values in the “Doña María” variety under direct exposure conditions (uncovered). In contrast, the “Aledo” variety showed a significantly lower concentration of this compound. As for stearic acid (C18:0), a notable differential behavior was observed: while in “Doña María” bagging caused a slight decrease, in “Aledo” the fruit protection treatment induced an increase of 16%. Other minor SFAs, such as arachidic acid (C20:0) and behenic acid (C22:0), also showed a strong dependence on the Variety × Treatment interaction (*p* < 0.001), with “Doña María” presenting consistently higher levels in almost all combinations.

With regard to MUFA, oleic acid (C18:1 c9/n9) represented the largest fraction in both varieties. Unlike what was observed in the previous season, no statistically significant differences were detected between “Doña María” and “Aledo” for this compound, nor was there a direct effect of the treatment in isolation. However, the interaction between both factors was significant (*p* < 0.05), showing that the impact of the microclimate of the bag on oleic synthesis depends strictly on the genotype cultivated. On the other hand, palmitoleic acid (C16:1) showed higher levels in the ‘Doña María’ variety compared to “Aledo”, confirming that this mid-season variety has a slightly higher MUFA accumulation capacity under the climatic conditions of 2023.

**Figure 2:**
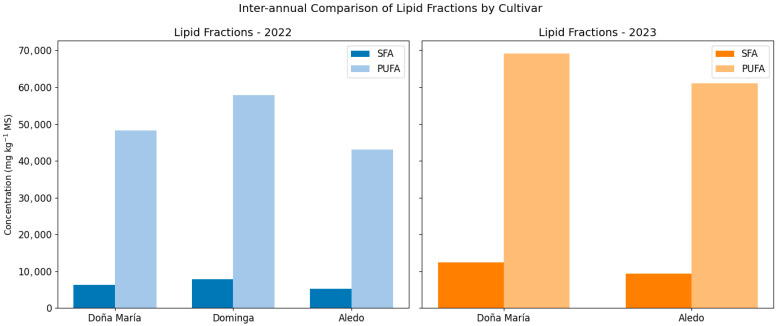
Inter-annual comparison of SFA and PUFA concentrations (2022 vs. 2023). Bar chart showing the significant increase in total lipid accumulation during the second productive cycle for ‘Doña María’, ‘Dominga’, and ‘Aledo’. Values represent the mean of three biological replicates (±SD); concentrations are expressed in mg kg^−1^ dry weight (DW).

Figure 2 illustrates the significant variations in the concentrations of SFAs and PUFAs between the two production cycles evaluated. A dramatic increase in total lipid accumulation was observed during the 2023 season compared to 2022, affecting all varieties analyzed. Specifically, in the “Doña María” cultivar, the concentration of PUFAs experienced a remarkable increase of more than 40%. A similar trend was observed in “Aledo,” where SFA levels nearly doubled in the second year.

This phenomenon of interannual lipid enrichment points toward a putative adaptive metabolic response of the berry to the specific climatic conditions of the Novelda area (Alicante). The increased synthesis of fatty acids—critical components of cell membranes—may represent a thermal protection mechanism against the more extreme summer temperatures recorded in 2023, potentially functioning to preserve cell integrity and modulate membrane fluidity during ripening. Furthermore, Figure 2 highlights that, although absolute concentrations fluctuated, the relative predominance of PUFAs over SFAs remained constant, ensuring the nutritional quality of the fruit across both seasons.

In the MUFA fraction (Table 5), significant variations were detected, influenced by all the factors analyzed. Palmitoleic acid (C16:1) showed clear superiority in the “Doña María” variety compared to “Aledo”. With regard to treatment, bagging favored a greater accumulation of this compound compared to exposed clusters. As for oleic acid (C18:1 c9/n9), although it is the most voluminous component of this fraction, the analysis of variance did not report statistically significant differences by variety or treatment in isolation, although the V × T interaction suggests a tendency for greater accumulation in the combination “Doña María” × uncovered. Likewise, eicosenoic acid (C20:1) presented significantly higher levels under the bagging treatment.

The PUFA fraction was qualitatively dominated by linoleic acid (C18:2). Significant differences (*p* < 0.05) were observed among cultivars, with “Doña María” reaching significantly higher concentrations than “Aledo”. Notably, the bagging treatment induced a significant accumulation of linoleic acid, presenting higher levels than the uncovered control. Regarding α-linolenic acid (C18:3), the “Doña María” variety exhibited levels almost twice as high as those found in ‘Aledo’ (*p* < 0.001). A finding of note was the tentative detection of a chromatographic peak matching the retention time of EPA, C20:5 n3 exclusively in the “Doña María” variety. Given that EPA is highly atypical in terrestrial higher plants, this signal must be interpreted with strict analytical caution. Using GC-FID methodology, it is highly probable that this peak represents a co-elution. The unique metabolic matrix of the “Doña María” cultivar likely produces a structurally similar long-chain lipid or a specific secondary metabolite that shares the exact retention time with the EPA standard under the applied chromatographic conditions. Therefore, while this signal serves as a distinct chemical fingerprint differentiating “Doña María” from “Dominga” and “Aledo”, definitive structural confirmation via Gas Chromatography–Mass Spectrometry (GC-MS) will be essential in future studies.

**Figure 3:**
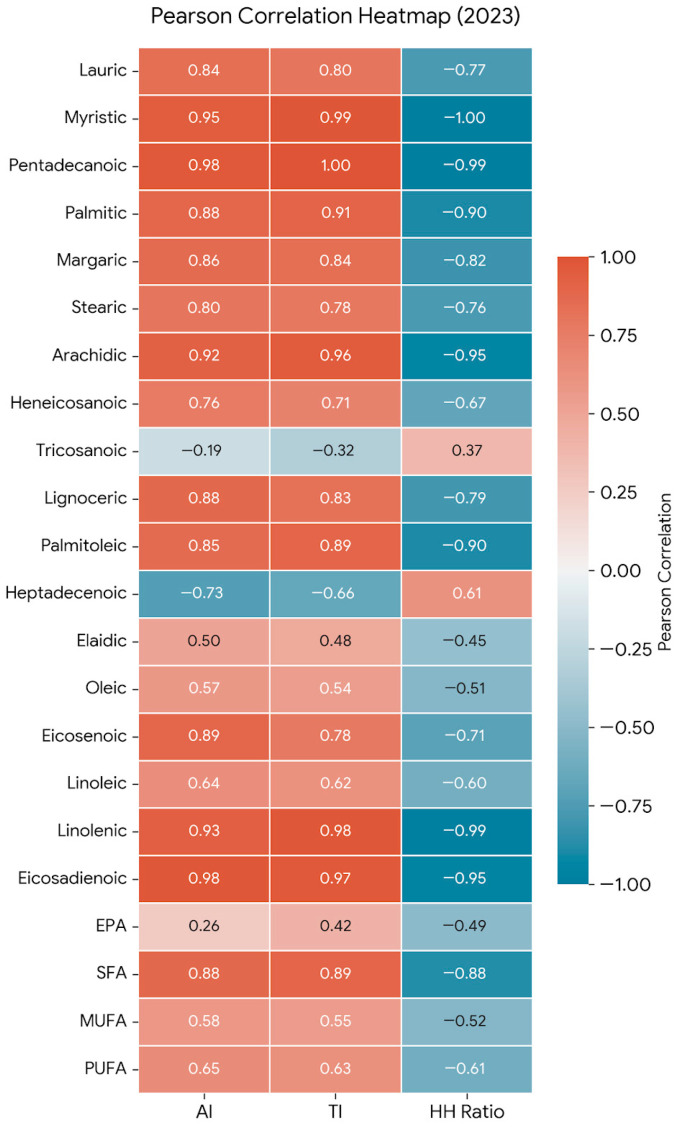
Pearson correlation heatmap between individual fatty acids and nutritional health indices (2023). Red tiles indicate a strong positive correlation, while blue tiles indicate a strong negative correlation (*p* < 0.05). The matrix highlights the statistical relationship between SFA (C16:0, C18:0) and pro-atherogenic indices (AI and TI).

**Figure 4:**
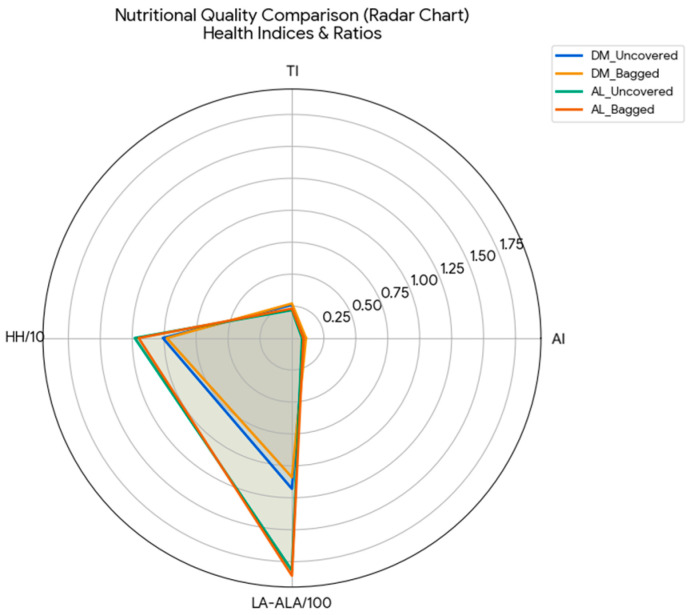
Nutritional Quality Radar Chart comparing cardiovascular health indices. The spider plot summarizes the functional potential of “Doña María” and “Aledo” under different treatments (uncovered vs. bagged). Indices include Atherogenicity Index (AI), Thrombogenicity Index (TI), Hypocholesterolemic/Hypercholesterolemic (HH) ratio, and the Linoleic/α-Linolenic (LA/ALA) ratio.

To visually confirm the weighting of the mathematical model proposed by Ulbricht and Southgate [25] within our specific matrix, a Pearson correlation analysis was performed mapping the primary fatty acids against the calculated functional indices (Figure 3). As expected mathematically since these fatty acids act as inputs in the equations a strong positive correlation (r > 0.90; *p* < 0.01) was observed between the major SFA (palmitic and stearic acids) and the pro-atherogenic indices (AI and TI). The value of this matrix, however, resides in highlighting the specific antagonic force exerted by the polyunsaturated fraction in table grapes. Linoleic acid (C18:2), being the dominant fatty acid, exhibits an inverse association that mathematically neutralizes the impact of the saturated fraction, maintaining the health indices at their optimal levels. Furthermore, Figure 4 illustrates that the bagging treatment does not alter the fundamental shape of the radar for any of the varieties, suggesting that the structural lipid proportion (SFA vs. PUFA) is a highly stable genetic trait under these agronomic conditions.

In contrast, the hypocholesterolemic/hypercholesterolemic (HH) ratio showed a robust negative correlation with the sum of SFA, validating its effectiveness as an indicator of the fruit’s ability to reduce plasma cholesterol. As for the polyunsaturated fraction, linoleic acid (C18:2) exhibited a positive correlation with total lipid load and an inverse association with the AI and TI indices. This interaction underscores that the high density of PUFA in varieties such as “Doña María” and “Aledo” acts as a protective factor that neutralizes the impact of SFA, maintaining health indices at optimal levels for human consumption.

In the 2023 campaign, total fatty acid concentrations (Table 6) showed a substantial increase compared to the previous cycle, with the ‘Doña María’ variety recording the highest values of saturated fatty acids and polyunsaturated fatty acids. The analysis of variance determined that bagging significantly increased the accumulation of SFA and PUFA compared to exposed bunches. The V X T interaction was particularly notable in the saturated fraction, where the combination ‘Doña María’ without bagging reached the absolute maximum, while ‘Aledo’ without bagging had the minimum value. In contrast, the monounsaturated fraction (∑ MUFA) showed no statistically significant differences between varieties or treatments.

Cardiovascular health indices confirmed the excellent nutritional quality of table grapes, despite the increase in absolute lipid concentration. The AI and TI remained at optimal levels, with no significant variations due to variety or cultural management. However, the Hypocholesterolemic/Hypercholesterolemic (HH) ratio and the LA/ALA ratio showed critical differences (*p* < 0.001). The ‘Aledo’ variety had a significantly higher HH ratio than ‘Doña María’, indicating a lipid profile with a greater capacity to reduce plasma cholesterol. Likewise, the LA/ALA ratio was considerably higher in ‘Aledo’ than in ‘Doña María’. The bagging treatment slightly reduced both parameters, with the HH ratio standing at 10.9 and the LA/ALA ratio at 147 in the protected clusters.

## 4. Discussion

When comparing the fatty acid profile of Vinalopó PDO table grapes with other *Vitis vinifera* cultivars, the dominance of linoleic acid (C18:2) emerges as a conserved chemotaxonomic trait. It must be noted that most of the existing literature focuses on extracted seed oils or winery by-products (pomace) rather than the whole berry. For instance, recent studies analyzing grape pomace from Southern Spain and Transylvania report linoleic acid as the predominant PUFA, followed by oleic and palmitic acids [8,27,28].

While the absolute lipid yield in our fresh table grapes is naturally lower than that of seed matrices, the structural proportions, specifically the high PUFA to SFA ratio, remain highly consistent. This confirms that the health-promoting lipid architecture is conserved across the species, although pre-harvest agronomic practices, such as bagging, exert a specific fine-tuning effect on the final accumulation in the edible portions of the fruit.

The marked interannual variability in fatty acid accumulation (2023 vs. 2022 season) reflects an adaptive response of the berry exocarp to environmental conditions. The PUFAs are not only energy reserves but also essential components of membranes that modulate cell fluidity in response to thermal stress.

The significant increase in PUFAs observed in 2023 suggests an adaptive lipid remodeling response to extreme thermal stress. Based on the existing literature, this phenomenon is often linked to the modulation of desaturase enzyme pathways, a mechanism documented in various crops grown in warm climates to prevent lipid peroxidation and maintain membrane fluidity [29]. Although our current study relies exclusively on metabolite profiling and lacks direct transcriptomic or enzymatic determination, these lipidomic shifts strongly support the hypothesis that heat stress triggers a protective biochemical cascade in table grapes.

Furthermore, a minor chromatographic peak tentatively matching EPA C20:5 was detected exclusively in unbagged ‘Doña María’ grapes. Given its trace concentration and negligible nutritional contribution and the inherent limitations of GC-FID identification, this signal likely represents a co-eluting compound or an exogenous artifact from the epicuticular microbiota, consistent with similar trace detections recently reported in grape pomace [8].

The visual comparison of nutritional quality indices, consolidated in the Radar Chart (Figure 4), allows us to identify the functional superiority of the cultivars analyzed compared to other vegetable lipid matrices. By projecting the Ulbricht and Southgate indices [25] together with the HH and LA/ALA ratios, it can be seen that the “Aledo” variety exhibits the most balanced profile from the point of view of cholesterol metabolism, occupying a significantly larger area on the HH axis compared to “Doña María”. Analysis of the spider chart reveals that, although “Doña María” has a higher absolute concentration of PUFA, it is “Aledo” that has a lipid architecture with lower relative atherogenic potential.

Moreover, the AI and TI values obtained for all Vinalopó PDO varieties are significantly lower than those reported for other vegetable oils and fruit by-products. For example, recent studies on organically grown grape seed oils show AI values ranging from 0.27 to 0.44 [30], whereas in other Mediterranean berries and nuts such as walnuts, these indices reflect greater variability depending on the cultivar [31,32]. The functional superiority observed in our samples, especially in the “Aledo” variety, highlights the value of bagged table grapes as a balanced source of fatty acids with an optimized n-6/n-3 ratio, comparable to high-quality lipid matrices reported in microalgae biotechnology and emerging seed oils [33].

Furthermore, Figure 4 illustrates that bagging treatment does not alter the fundamental shape of the radar for any of the varieties, suggesting that functional quality is a resilient genetic trait. However, the slight contraction of the radar area in the protected samples of “Doña María” reflects the sensitivity of omega-3 fatty acids to the microclimate of the bag, a finding that should be considered when assessing the added nutritional value of bagged grapes versus direct exposure.

Beyond their structural and thermal adaptation functions, PUFAs, especially linoleic acid and α-linolenic acid, play a crucial physiological role as primary precursors of the volatile compounds that define the aroma of grapes [34,35]. During ripening and the post-harvest stage, these lipids act as direct substrates of the lipoxygenase (LOX) pathway, an enzymatic cascade that oxidizes and cleaves them to form aldehydes, alcohols, and six-carbon (C6) esters, responsible for the green and fruity sensory notes so highly valued in table grapes [36,37].

Recent transcriptomic studies on other *Vitis vinifera* varieties confirm that microclimate changes can modulate the expression of key lipoxygenase pathway genes (such as VvLOX and VvHPL), which regulate the conversion of PUFAs into aromatic compounds [38,39]. While our methodology focused on quantifying the structural lipid substrates rather than real-time enzymatic activity, the significant variability observed in linoleic acid levels between “Doña María” and “Aledo” grapes under bagging provides a robust metabolic foundation to explain the differences in their aromatic profiles. This positions fatty acid profiling as an integral, albeit indirect, marker of the fruit’s organoleptic potential [40].

**Figure 5:**
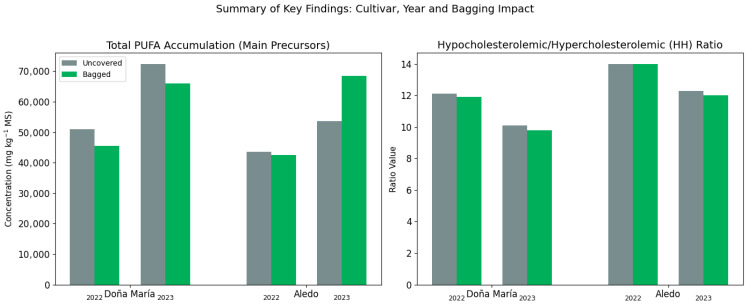
Summary of key findings: The impact of cultivar, campaign, and bagging on table grape quality. Schematic representation of the interplay between environmental factors and the lipid reserve. The diagram highlights the preservation of nutritional quality and the modulation of fatty acid precursors for the lipoxygenase (LOX) pathway.

Figure 5 summarizes the biological model proposed for table grapes from the Vinalopó PDO, integrating the findings from the 2022 and 2023 campaigns. The study reveals that lipid accumulation in the exocarp is not a static process, but rather a dynamic response modulated by the interaction between genotype and the microclimate induced by bagging. While the genotype dictates the maximum endogenous health potential (such as the higher HH ratio in “Aledo”), interannual conditions act as a metabolic switch that can double the reserve of PUFAs.

The fluctuation observed in the lipid profile between the 2022 and 2023 seasons highlights the metabolic plasticity of table grapes in response to climate change. The significant increase in PUFAs in 2023, a year characterized by extreme summer temperatures in southeastern Spain, suggests an adaptation mechanism to counteract heat stress and preserve the fluidity of the exocarp membranes [41]. Recent studies indicate that the stability of fatty acids under moderate or extreme thermal conditions is a determining factor in the post-harvest quality and shelf life of fruit [21,42].

Likewise, the role of lipids as precursors of defense signals against abiotic stress is fundamental, since lipid remodeling not only affects cell structure, but also modulates the berry’s response to solar radiation and drought [43,44]. This metabolic resilience, observed especially in bagged varieties, confirms that the microclimate generated by the bag interacts with the macroclimatic conditions of the year, allowing for a differential accumulation of bioactive compounds that act as natural thermal protectors [45,46].

This metabolic flow, summarized in Figure 5, highlights that bagging preserves the integrity of the precursors of the lipoxygenase (LOX) pathway. The physical protection of the cluster not only guarantees superior aesthetics, but also maintains a lipid substrate of high nutritional quality which, after enzymatic degradation, will give rise to the volatiles responsible for the fruit’s characteristic aroma. Ultimately, the model confirms that bagged table grapes represent an optimal balance between crop protection and the biosynthesis of bioactive compounds, consolidating their status as a functional food resilient to climate variability.

While the two-year temporal scope of this study captured a highly significant climatic contrast, it inherently presents limitations for modeling long-term lipidomic trends. As contextualized by the 30-year historical climatic baseline, the exceptionality of the 2023 season provided a unique natural experiment to observe acute metabolic plasticity under severe thermal and drought stress. However, extending the temporal scope through future longitudinal field studies, spanning multiple standard and atypical seasons, will be essential to fully disentangle natural interannual variability from chronic climate change adaptations in the table grape lipidome.

## 5. Conclusions

This study demonstrates that the lipid profile of Vinalopó PDO table grapes is significantly modulated by the interaction between genotype, interannual climatic conditions, and pre-harvest bagging techniques. Qualitatively, all analyzed varieties exhibited a profile dominated by PUFAs, specifically linoleic acid, followed by MUFAs such as oleic acid. From a nutritional perspective, the atherogenicity (AI) and thrombogenicity (TI) indices remained at exceptionally low levels across both campaigns, reflecting a favorable lipid architecture. The ‘Aledo’ variety consistently exhibited the highest hypocholesterolemic/hypercholesterolemic (HH) ratio, highlighting its distinct functional value in terms of lipid balance.

The bagging treatment showed a dual metabolic behavior dependent on the production cycle. While in 2022 it tended to reduce total lipid accumulation, in 2023 it favored a higher concentration of SFAs and PUFAs. This variability suggests that the paper bag acts as a thermal and light regulator that may modulate fatty acid desaturase activity differentially according to environmental stress. In conclusion, bagging not only provides physical protection but also preserves a lipid profile rich in aromatic precursors, ensuring nutritional stability in the edible portion of the fruit.

Future research should focus on integrating transcriptomic analyses to validate the specific regulation of desaturase and lipoxygenase (LOX) pathway genes under extreme heat stress. Additionally, long-term post-harvest studies are needed to determine how these heat-induced lipidomic shifts directly impact the shelf-life, sensory profile, and overall resilience of table grapes during storage and cold chain distribution.

From a practical standpoint, these findings provide viticulturists and the table grape industry with actionable biochemical markers. Understanding how bagging and microclimate variations dynamically modulate the lipid profile allows for optimized pre-harvest management and targeted harvest timing, ultimately empowering the production chain to standardize the nutritional value and aromatic quality of PDO table grapes in an era of increasing climate volatility.

## Figures and Tables

**Table 1:** Saturated fatty acids and monounsaturated fatty acids composition (mg kg^−1^) in PDO Vinalopó table grape cultivars under different bagging treatments in 2022.

	SFA								MUFA				
	Myristic	Pentadecanoic	Palmitic	Margaric	Stearic	Arachidic	Behenic	Tricosanoic	Palmitoleic	Heptadecenoic	Elaidic	Oleic	Erucic
	ANOVA Test ^†^												
Variety	**	*	*	NS	***	***	***	NS	NS	*	NS	*	***
Treatment	**	NS	*	NS	***	***	NS	NS	**	*	NS	*	NS
Variety × Treatment	**	NS	*	NS	***	***	***	NS	**	*	NS	*	***
	Tukey Multiple Range Test ^‡^												
Variety													
Doña María	29.8 ^a^	6.13 ^a^	4880 ^ab^	17.8	1304 ^b^	36.6 ^b^	nd ^c^	4.52	83.8	7.19 ^a^	9.77	10,488 ^b^	8.90 ^a^
Dominga	27.1 ^a^	5.40 ^ab^	5153 ^a^	15.8	2518 ^a^	43.2 ^a^	2.75 ^b^	5.33	68.8	5.25 ^ab^	9.56	14,052 ^a^	nd ^b^
Aledo	24.6 ^b^	4.34 ^b^	3927 ^b^	17.2	1252 ^b^	20.4 ^c^	4.59 ^a^	3.89	71.7	4.31 ^b^	6.82	12,344 ^ab^	nd ^b^
Treatment													
Uncovered	29.3 ^a^	5.45	5023 ^a^	19.4	1867 ^a^	38.4 ^a^	2.77	4.86	81.1 ^a^	6.75 ^a^	9.80	13,349 ^a^	2.34
Bagged	24.9 ^b^	5.13	4285 ^b^	14.5	1516 ^b^	28.4 ^b^	2.12	4.30	68.5 ^b^	4.41 ^b^	7.63	11,240 ^b^	3.59
Variety × Treatment													
Doña Maria × Bagged	18.7 ^b^	5.82	4683 ^ab^	17.1	1215 ^c^	31.6 ^bc^	nd ^c^	3.49	76.6 ^ab^	5.29 ^ab^	8.02	10,205 ^b^	10.8 ^a^
Doña Maria × Uncovered	40.9 ^a^	6.44	5077 ^ab^	18.6	1392 ^c^	41.7 ^ab^	nd ^c^	5.54	91.1 ^a^	9.08 ^a^	11.5	10,770 ^b^	7.01 ^a^
Dominga × Bagged	31.0 ^ab^	4.94	4265 ^ab^	10.6	2080 ^b^	35.2 ^b^	1.58 ^bc^	4.86	58.5 ^b^	3.22 ^b^	8.07	10,819 ^b^	nd ^b^
Dominga × Uncovered	23.1 ^b^	5.85	6041 ^a^	21.1	2955 ^a^	51.3 ^a^	3.93 ^ab^	5.80	79.1 ^ab^	7.27 ^ab^	11.0	17,285 ^a^	nd ^b^
Aledo × Bagged	25.2 ^b^	4.62	3906 ^b^	15.7	1252 ^c^	18.5 ^d^	4.79 ^a^	4.55	70.3 ^ab^	4.72 ^ab^	6.79	12,697 ^ab^	nd ^b^
Aledo × Uncovered	23.9 ^b^	4.06	3950 ^b^	18.6	1253 ^c^	22.3 ^cd^	4.39 ^a^	3.24	73.1 ^ab^	3.89 ^ab^	6.85	11,991 ^ab^	nd ^b^

† NS = not significant at *p* > 0.05; *, **, ***, significant at *p* < 0.05, 0.01, and 0.001, respectively. ‡ Values (mean of three replications) followed by the same letter within the same column are not significantly different (*p* > 0.05) according to Tukey’s least significant difference test. nd = not detected (concentration below the limit of detection). Fatty acid nomenclature: Myristic (C14:0); Pentadecanoic (C15:0); Palmitic (C16:0); Margaric (C17:0); Stearic (C18:0); Arachidic (C20:0); Behenic (C22:0); Tricosanoic (C23:0); Palmitoleic (C16:1 n-7); Heptadecenoic (C17:1); Elaidic (C18:1 t-9); Oleic (C18:1 n-9); Erucic (C22:1 n-9). Regarding the quantitative impact on the lipid reserve, the bagging treatment led to a slight decrease in the total lipid yield across the studied cultivars. On average, bagged grapes showed a 14.0% lower total fatty acid content compared to uncovered samples (averaging 63,400 mg kg^−1^ vs. 73,700 mg kg^−1^). This quantitative shift, although secondary to the profile quality changes, suggests that the modified microclimate inside the bag also influences the overall rate of triacylglycerol assembly and lipid storage in the mesocarp.

**Table 2:** Polyunsaturated fatty acids composition (mg kg^−1^) in PDO Vinalopó table grape cultivars under different bagging treatments in 2022.

	PUFA				
	Linolelaidic	Linoleic	α-Linolenic	Eicosadienoic	EPA
	ANOVA Test ^†^				
Variety	NS	*	***	NS	***
Treatment	NS	*	***	NS	***
Variety × Treatment	NS	*	***	NS	***
	Tukey Multiple Range Test ^‡^				
Variety					
Doña María	0.71	47,740 ^ab^	370 ^a^	8.68	tr
Dominga	0.00	57,593 ^a^	299 ^b^	4.76	nd
Aledo	0.00	42,797 ^b^	201 ^c^	4.48	nd
Treatment					
Uncovered	0.00	52,849 ^a^	312 ^a^	7.21	tr
Bagged	0.47	45,904 ^a^	268 ^b^	4.74	nd
Variety × Treatment					
Doña Maria × Bagged	1.42	45,152 ^ab^	349 ^ab^	5.68	nd
Doña Maria × Uncovered	0.00	50,329 ^ab^	391 ^a^	11.68	tr
Dominga × Bagged	0.00	50,316 ^ab^	258 ^bc^	4.07	nd
Dominga × Uncovered	0.00	64,871 ^a^	341 ^ab^	5.45	nd
Aledo × Bagged	0.00	42,246 ^b^	197 ^c^	4.46	nd
Aledo × Uncovered	0.00	43,348 ^b^	205 ^c^	4.50	nd

† NS = not significant at *p* > 0.05; *, ***, significant at *p* < 0.05 and 0.001, respectively. ‡ Values (mean of three replications) followed by the same letter within the same column are not significantly different (*p* > 0.05) according to Tukey’s least significant difference test. nd = not detected. tr = trace concentration. Tentative identification due to potential co-elution; absolute quantification not reliable. Fatty acid nomenclature: Linolelaidic (C18:2 trans n-6); Linoleic (C18:2 n-6); α-Linolenic (C18:3 n-3); Eicosadienoic (C20:2 n-6); EPA (C20:5 n-3).

**Table 3:** Total Fatty acid composition (mg kg^−1^), Index of atherogenicity (AI), Index of thrombogenicity (TI), Polyunsaturated Fatty Acid/Saturated Fatty Acid (PUFA/SFA) and Hypocholesterolemic/hypercholesterolemic ratio (HH) and Linoleic acid/α-linolenic acid ratio (LA/ALA) ratio in PDO Vinalopó table grape cultivars under different bagging treatments in 2022.

	ƩSFA	ƩMUFA	ƩPUFA	AI	TI	ΣPUFA/ΣSFA	HH	LA/ALA
	ANOVA Test ^†^							
Variety	**	*	*	NS	NS	NS	NS	***
Treatment	**	*	*	NS	NS	NS	NS	NS
Variety × Treatment	**	*	*	NS	NS	NS	NS	***
	Tukey Multiple Range Test ^‡^							
Variety								
Doña María	6279 ^ab^	10,597 ^b^	48,292 ^ab^	0.09	0.20	7.69	12.0	130 ^c^
Dominga	7771 ^a^	14,136 ^a^	57,897 ^a^	0.07	0.21	7.51	14.0	193 ^b^
Aledo	5,255 ^b^	12,427 ^ab^	43,003 ^b^	0.07	0.19	8.16	14.0	212 ^a^
Treatment								
Uncovered	6990 ^a^	13,449 ^a^	53,262 ^a^	0.08	0.20	7.71	13.2	176
Bagged	5880 ^b^	11,324 ^b^	46,200 ^b^	0.08	0.20	7.87	13.4	180
Variety × Treatment								
Doña Maria × Bagged	5975 ^b^	10,306. ^b^	45,574 ^ab^	0.09	0.20	7.63	11.90	130 ^c^
Doña Maria × Uncovered	6582 ^ab^	10,889 ^b^	51,011 ^ab^	0.09	0.20	7.76	12.10	129 ^c^
Dominga × Bagged	6434 ^ab^	10,889 ^b^	50,578 ^ab^	0.07	0.20	7.86	14.30	195 ^ab^
Dominga × Uncovered	9107 ^a^	17,383 ^a^	65,217 ^a^	0.07	0.21	7.16	13.60	190 ^b^
Aledo × Bagged	5231 ^b^	12,778 ^ab^	42,448 ^b^	0.07	0.18	8.12	14.00	214 ^a^
Aledo × Uncovered	5280 ^b^	12,075 ^ab^	43,558 ^b^	0.07	0.19	8.20	14.00	210 ^ab^

† NS = not significant at *p* > 0.05; *, **, ***, significant at *p* < 0.05, 0.01, and 0.001, respectively. ‡ Values (mean of three replications) followed by the same letter within the same column are not significantly different (*p* > 0.05) according to Tukey’s least significant difference test.

**Table 4:** Saturated fatty acids composition (mg kg^−1^) in PDO Vinalopó table grape cultivars under different bagging treatments in 2023.

	SFA									
	Lauric	Myristic	Pentadecanoic	Palmitic	Margaric	Stearic	Arachidic	Heneicosanoic	Tricosanoic	Lignoceric
	ANOVA Test ^†^									
Variety	*	***	***	**	*	*	**	NS	**	***
Treatment	*	***	***	**	*	*	**	NS	**	***
Variety × Treatment	*	***	***	**	*	*	**	NS	**	***
	Tukey Multiple Range Test ^‡^									
Variety										
Doña María	37.5 ^a^	79.7 ^a^	26.5 ^a^	8779 ^a^	55.5 ^a^	3271 ^a^	142 ^a^	29.2	1.54 ^b^	51.1 ^a^
Aledo	21.3 ^b^	42.7 ^b^	14.5 ^b^	6420 ^b^	46.4 ^b^	2699 ^b^	95.8 ^b^	23.1	5.31 ^a^	25.3 ^b^
Treatment										
Uncovered	24.5 ^b^	58.4 ^b^	19.5 ^b^	7314 ^b^	48.8 ^b^	2841 ^b^	114 ^b^	23.8	1.54 ^b^	28.7 ^b^
Bagged	34.3 ^a^	64.0 ^a^	22.0 ^a^	7886 ^a^	53.1 ^a^	3129 ^a^	123 ^a^	28.6	5.31 ^a^	47.7 ^a^
Variety × Treatment										
Doña Maria × Uncovered	26. ^ab^	78.5 ^a^	25.1 ^a^	9061 ^a^	56.4 ^a^	3364 ^a^	145 ^a^	30.0	3.07 ^ab^	33.6 ^ab^
Doña Maria × Bagged	48.1 ^a^	80.8 ^a^	27.9 ^a^	8498 ^a^	54.7 ^a^	3178 ^a^	140 ^ab^	28.5	0.00 ^b^	68.7 ^a^
Aledo × Uncovered	22.1 ^ab^	38.2 ^b^	13.0 ^b^	5566 ^b^	41.1 ^b^	2318 ^b^	83.8 ^c^	17.5	0.00 ^b^	23.8 ^b^
Aledo × Bagged	21.0 ^b^	47.2 ^b^	16.1 ^b^	7275 ^ab^	51.6 ^ab^	3080 ^ab^	108 ^bc^	28.6	10.6 ^a^	26.7 ^b^

† NS = not significant at *p* > 0.05; *, **, ***, significant at *p* < 0.05, 0.01, and 0.001, respectively. ‡ Values (mean of three replications) followed by the same letter within the same column are not significantly different (*p* > 0.05) according to Tukey’s least significant difference test. Fatty acid nomenclature: Lauric (C12:0); Myristic (C14:0); Pentadecanoic (C15:0); Palmitic (C16:0); Margaric (C17:0); Stearic (C18:0); Arachidic (C20:0); Heneicosanoic (C21:0); Tricosanoic (C23:0); Lignoceric (C24:0).

**Table 5:** Monounsaturated fatty acids and polyunsaturated fatty acids composition (mg kg^−1^) in PDO Vinalopó table grape cultivars under different bagging treatments in 2023.

	MUFA					PUFA			
	Palmitoleic	Heptadecenoic	Elaidic	Oleic	Eicosenoic	Linoleic	α-Linolenic	Eicosadienoic	EPA
	ANOVA Test ^†^								
Variety	**	NS	NS	NS	**	*	***	NS	*
Treatment	**	NS	NS	NS	**	*	***	NS	*
Variety × Treatment	**	NS	NS	NS	**	*	***	NS	*
	Tukey Multiple Range Test ^‡^								
Variety									
Doña María	127 ^a^	7.87	23.2	19,290	11.2 ^a^	68,502 ^a^	607 ^a^	35.5	tr
Aledo	87.3 ^b^	10.2	20.0	17,682	8.64 ^b^	60,691 ^b^	331 ^b^	26.9	nd
Treatment									
Uncovered	104 ^b^	10.2	20.5	17,918	8.32 ^b^	62,496 ^b^	453 ^b^	29.4	tr
Bagged	110 ^a^	7.83	22.7	19,054	11.5 ^a^	66,697 ^a^	486 ^a^	33.0	nd
Variety × Treatment									
Doña Maria × Uncovered	134 ^a^	7.72	25.5	20,100	10.0 ^ab^	71,725 ^a^	612 ^a^	32.9	tr
Doña Maria × Bagged	120 ^ab^	8.02	21.0	18,481	12.4 ^a^	65,279 ^ab^	602 ^a^	38.2	nd
Aledo × Uncovered	74.4 ^c^	12.7	15.4	15,736	6.62 ^b^	53,266 ^b^	294 ^b^	25.9	nd
Aledo × Bagged	100 ^bc^	7.64	24.5	19,628	10.67 ^a^	68,116 ^ab^	369 ^b^	27.8	nd

† NS = not significant at *p* > 0.05; *, **, ***, significant at *p* < 0.05, 0.01, and 0.001, respectively. ‡ Values (mean of three replications) followed by the same letter within the same column are not significantly different (*p* > 0.05) according to Tukey’s least significant difference test. nd = not detected. tr = trace concentration. Tentative identification due to potential co-elution; absolute quantification not reliable. Fatty acid nomenclature: Palmitoleic (C16:1 n-7); Heptadecenoic (C17:1); Elaidic (C18:1 t-9); Oleic (C18:1 n-9); Eicosenoic (C20:1 n-9); Linoleic (C18:2 n-6); α-Linolenic (C18:3 n-3); Eicosadienoic (C20:2 n-6); EPA (C20:5 n-3).

**Table 6:** Total Fatty Acid composition (mg kg^−1^), Index of atherogenicity (AI), Index of thrombogenicity (TI), Polyunsaturated Fatty Acid/Saturated Fatty Acid (PUFA/SFA) and Hypocholesterolemic/hypercholesterolemic ratio (HH) and Linoleic acid/α-linolenic acid ratio (LA/ALA) in PDO Vinalopó table grape cultivars under different bagging treatments in 2023.

	ƩSFA	ƩMUFA	ƩPUFA	AI	TI	ΣPUFA/ΣSFA	HH	LA/ALA
	ANOVA Test ^†^							
Variety	**	NS	*	NS	NS	NS	***	***
Treatment	**	NS	*	NS	NS	NS	***	***
Variety × Treatment	**	NS	*	NS	NS	NS	***	***
	Tukey Multiple Range Test ^‡^							
Variety								
Doña María	12,474 ^a^	19,460	69,147 ^a^	0.10	0.27	5.54	9.94 ^b^	113 ^b^
Aledo	9394 ^b^	17,808	61,049 ^b^	0.08	0.23	6.52	12.2 ^a^	184 ^a^
Treatment								
Uncovered	10,474 ^b^	18,062	62,980 ^b^	0.09	0.24	6.13	11.2 ^a^	150 ^a^
Bagged	11,394 ^a^	19,206	67,216 ^a^	0.10	0.25	5.93	10.9 ^b^	147 ^b^
Variety × Treatment								
Doña Maria × Uncovered	12,823 ^a^	20,278	72,376 ^a^	0.10	0.26	5.65	10.1 ^b^	118 ^b^
Doña Maria × Bagged	12,124 ^a^	18,642	65,919 ^ab^	0.11	0.27	5.43	9.78 ^b^	109 ^b^
Aledo × Uncovered	8124 ^b^	15,846	53,585 ^b^	0.08	0.22	6.61	12.3 ^a^	182 ^a^
Aledo × Bagged	10,664 ^ab^	19,771	68,513 ^ab^	0.09	0.23	6.43	12.0 ^a^	186 ^a^

† NS = not significant at *p* > 0.05; *, **, ***, significant at *p* < 0.05, 0.01, and 0.001, respectively. ‡ Values (mean of three replications) followed by the same letter within the same column are not significantly different (*p* > 0.05) according to Tukey’s least significant difference test.

## Data Availability

All data generated and analyzed during this study are included in this published article. Additional information is available from the corresponding author upon request.

## Supplementary Materials

The following supporting information can be downloaded at: https://www.mdpi.com/article/10.3390/foods15091585/s1, Table S1. Loadings matrix of the Principal Component Analysis (PCA) for the fatty acid profile of Vinalopó PDO table grapes (2023 campaign).

## Abbreviations

The following abbreviations are used in this manuscript:

AI	Atherogenicity Index
ALA	α-Linolenic Acid
DW	Dry Weight
EPA	Eicosapentaenoic Acid
FAMEs	Fatty Acid Methyl Esters
GC-FID	Gas Chromatography-Flame Ionization Detector
HH	Hypocholesterolemic/Hypercholesterolemic ratio
LA	Linoleic Acid
LC-PUFA	Long-Chain Polyunsaturated Fatty Acid
LOX	Lipoxygenase
MUFA	Monounsaturated Fatty Acid
PCA	Principal Component Analysis
PDO	Protected Designation of Origin
PUFA	Polyunsaturated Fatty Acid
SFA	Saturated Fatty Acid
TI	Thrombogenicity Index
**UV**	Ultraviolet
