# Supplementary material for: Fatty Acid Profile of Table Grapes: Impact of Cultivar and Fruit Protection on Saturated, Monounsaturated, and Polyunsaturated Fatty Acid Profile
Source: Foods. 2026 May 4;15(9):1585. doi: 10.3390/foods15091585 (PMC13163263; doi:10.3390/foods15091585)
Supplement: Supplementary file 1 [file foods-15-01585-s001.zip › foods-4235484-supplementary.pdf]

Supplementary Table S1. Loadings matrix of the Principal Component Analysis (PCA) for the fatty acid profile of Vinalopó PDO table grapes (2023 campaign).

Fatty Acid	PC1 (Original)	PC2 (Original)	PC1 (Scaled)	PC2 (Scaled)
**C12:0**	-0.115	0.738	-1.5	7.2
**C14:0**	-0.040	0.770	-2.8	-2.5
**C15:0**	-0.070	0.774	-2.5	-3.5
**C16:0**	-0.256	0.251	-3.3	2.4
**C16:1**	-0.220	0.537	-2.9	5.2
**C17:0**	-0.248	0.215	-3.2	2.1
**C17:1**	0.208	-0.733	2.7	-7.2
**C18:0**	-0.250	0.234	-3.2	2.3
**C18:1c**	-0.273	-0.268	-2.8	1.5
**C18:1t**	-0.212	-0.618	-2.5	1.2
**C18:2c**	-0.273	-0.268	-3.5	-2.6
**C18:3**	-0.239	0.417	-2.5	-1.5
**C20:0**	-0.240	0.275	-3.1	2.7
**C20:1**	-0.185	0.405	-2.2	0.8
**C20:2**	-0.210	0.118	-2.7	1.2
**C21:0**	-0.199	0.449	-3.8	1.8
**C23:0**	-0.184	-0.726	-2.4	-7.1
**C24:0**	-0.188	0.464	-2.5	-4.5

Note: 'Original' columns represent the exact eigenvectors (loadings) extracted from the PCA, detailing the mathematical weight of each variable. 'Scaled' columns represent the coordinates used for the graphical projection in the biplot (Figure 1)
